# Antibiotic exposure among young infants suffering from diarrhoea in Bangladesh

**DOI:** 10.1111/jpc.15233

**Published:** 2020-10-27

**Authors:** Md Ridwan Islam, Sharika Nuzhat, Shah Mohammad Fahim, Parag Palit, Robin L Flannery, David J Kyle, Mustafa Mahfuz, M Munirul Islam, Shafiqul Alam Sarker, Tahmeed Ahmed

**Affiliations:** ^1^ Nutrition and Clinical Services Division International Centre for Diarrhoeal Disease Research, Bangladesh Dhaka Bangladesh; ^2^ Evolve BioSystems, Inc. Davis California United States

**Keywords:** antibiotic, diarrhoea, infant, malnutrition

## Abstract

**Aims:**

Appropriate rehydration has always been significant in treating diarrhoeal diseases in children. Irrational antibiotic use among diarrhoeal children has remained a major public health concern. Information regarding antibiotic use in young infants suffering from diarrhoea is very limited and a unique aspect of research. We aimed to investigate the prevalence of antibiotic use in the community among 2–6 months infants with diarrhoeal illnesses and having different nutritional status.

**Methods:**

We investigated a total of 5279 infants aged 2–6 months at Dhaka hospital, International Centre for Diarrhoeal Disease Research, Bangladesh, between September 2018 and June 2019. Among them, 257 infants were suffering from severe acute malnutrition (SAM). History of taking antibiotics was ascertained by direct observation of a prescription by a physician, the bottle of antibiotic or asking the caregiver about the name of antibiotic or its price that is very close to the usual market price of an antibiotic.

**Results:**

Overall, 52% of infants received antibiotics before hospital admission. Non‐SAM infants had higher odds of receiving antibiotics (adjusted odds ratio [aOR] = 1.52, 95% confidence interval: 1.18, 1.97, *P* value = 0.003) compared to infants with SAM and use of antibiotics increased with age (aOR = 1.11, 95% confidence interval: 1.06, 1.17, *P* value<0.001). Commonly used antibiotics were azithromycin (13.3%), ciprofloxacin (7.7%), erythromycin (7.7%) and metronidazole (2.6%). The proportion of receiving ciprofloxacin was significantly lower in infants with SAM compared to their non‐SAM counterparts (2.7% vs*.* 7.97%, *P* value = 0.004).

**Conclusions:**

The study underscores the excessive use of antibiotics among diarrhoeal infants, which is already a major public health concern in low‐ and middle‐income countries.

## What is already known on this topic

1. Irrational use of antibiotics in the management of diarrhoeal diseases has been a long‐standing public health burden globally including South Asian countries.
2. Antimicrobial resistance is increasing rapidly in South Asian regions, which is associated with the excessive use of antibiotics.
3. There is limited information regarding antibiotic exposure in young Bangladeshi infants suffering from acute watery diarrhoea.

## What this paper adds

1. A large proportion of young infants are being exposed to antibiotics at a very early age who were suffering from diarrhoea.
2. Antibiotic consumption was higher in infants without severe acute malnutrition in contrast to those with severe acute malnutrition.
3. Consumption of macrolides and fluoroquinolones was considerably elevated amidst this vulnerable age group.

Diarrhoea, which is recognised as a global burden, causes 440 000 child deaths world‐wide and is one of the leading causes of death among children aged less than 5 years.^1^ The suffering vastly resides in lower socio‐economic populations but people from higher socio‐economic status do share their part of the burden.^1^ The aetiology of diarrhoeal diseases is not limited to bacteria or parasites only, but virus also plays a major role in the burden of this illness. Different studies from diarrhoea endemic regions show that enteric viruses are responsible for a considerable amount of diarrhoeal episodes in children.^2^ For example; rotavirus was the leading cause for all diarrhoeal deaths among children below 5 years of age with more than 125 000 deaths globally.^1^ The Global Enteric Multicenter Study found that rotavirus was the main cause of acute diarrhoea among infants in all seven of their sites.^3^ When it comes to treating diarrhoea, antibiotic is not always the answer. Using antibiotics irrationally without proper justification is wrong as most of the diarrhoeal cases resolve spontaneously.^4^ Nearly 50% of patients who are suffering from diarrhoea get better within 3 days.^5^ Antimicrobials are only indicated in bloody diarrhoea and severe cholera and not to be ingested irrationally against general acute watery diarrhoea cases.^6^ Also, in the case of viral diarrhoea antibiotic does not play any role in wiping out the causative organism. Fluid and electrolyte replacement as well as zinc therapy have always been central to the case management of diarrhoeal disease.^4^

In a low socio‐economic setting, financial conditions and scarcity of diagnostic resources combined with the huge burden of the disease make it difficult for the patients to find out the causative organisms. As a result, antimicrobials are prescribed empirically, which is not justified.^7^ A study in Vietnam showed that antimicrobials were prescribed in 38% cases of watery diarrhoea, whence it was caused by viruses and in 60% of cases it was administered without knowing the actual aetiology.^8^ Unnecessary use of antimicrobials in humans as well as in animals in Southeast Asia has brought about this emerging crisis known as antimicrobial resistance (AMR) and it is increasing day by day against many first‐line antibiotics like third‐generation cephalosporin and fluoroquinolones across this region.^9^

Severe acute malnutrition (SAM) is recognised as a major global public health burden that threatens the lives of more than 14 million children under 5 years of age world‐wide.^10^ A significant proportion of children with this affliction reside in South Asian region among which Bangladesh holds a crucial position with more than 0.3 million children suffering from this acute form of malnutrition.^11^ Diarrhoea along with malnutrition complicates the overall recovery process for young children and case fatality rates for SAM children remain much higher than overall case fatality rates.^12^ Antibiotic using patterns can be considered vital during the course of the management among children especially infants. Antibiotic consumption in early life is also associated with dysbiosis of gut microbiota which can attribute to malnutrition and other acute and chronic illnesses.^13^, ^14^, ^15^ In light of the discussed issues, our aim was to investigate the prevalence of antibiotic use before hospital admission among 2–6 months infants with diarrhoeal illnesses and its association with different nutritional status.

## Methods

### Procedure

Data were extracted from the screening data set of a study entitled ‘Pilot of a Prebiotic and Probiotic Trial in Young Infants with Severe Acute Malnutrition’ (NCT03666572). A total of 5279 infants, aged 2–6 months, were screened between September 2018 and June 2019 when the infants were brought at Dhaka Hospital, International Centre for Diarrhoeal Disease Research, Bangladesh, for management of diarrhoeal illnesses. Dhaka hospital admits diarrhoeal children to the out‐patient department, short stay unit, longer stay unit and intensive care unit as required by the clinical condition of the patients. All infants were admitted to the hospital irrespective of their dehydration status and later sent to different units of the hospital after assessment and data collection. The patients were sick enough to warrant admission to the respective units of the hospital as required.

A total of 257 infants were suffering from SAM in our study. SAM was defined in less than 6 months of age by using World Health Organization (WHO) classification criteria. Weight‐for‐length less than −3 Z‐score and presence of bilateral pitting oedema were the two identifying points.^16^ Birth history of the infants depicting preterm birth, low birthweight and gestation corrected age were not used in classifying SAM in the study. Ninety‐nine infants were suffering from bilateral pedal oedema, which was nutritional in origin and was diagnosed following Integrated Management of Childhood Illness guidelines.^17^ Trained physicians working in the malnutrition unit diagnosed nutritional oedema cases. The patients were examined clinically by the physicians for any signs of congenital cardiac disease.

The participants' caregivers were asked if the infants consumed antibiotics in order to treat their present diarrhoeal disease for which they are seeking medical attention. The history of antibiotic usage was assessed by verification of a prescription by a registered physician, or showing the bottle that contained the antibiotic or asking the caregiver about the name of antibiotic or its price that is very close to the usual market price of an antibiotic. Morbidity and nutritional status were evaluated by physicians and trained health‐care workers immediately upon arrival of the patients at the hospital. Different clinical parameters were assessed clinically like septic shock, jaundice, Down's syndrome, presence of Anuria by a registered physician at the time of screening. Screening data were analysed anonymously, and this activity has the approval from both the Research Review Committee and the Ethical Review Committee of International Centre for Diarrhoeal Disease Research, Bangladesh.

### Definition

*Severe acute malnutrition (SAM)*: Weight‐for‐length Z‐score value less than −3 SD of WHO growth standards or presence of bilateral pedal oedema.^16^

*Not severe acute malnutrition (non‐SAM)*: Weight‐for‐length Z‐score value greater than − 3 SD of WHO growth standards.

### Data analysis

Data were analysed using SPSS for Windows (version 20; SPSS Inc., Chicago, IL, USA). χ^2^ test or Fisher's exact test were applied for comparison between the categorical variables. Student's *t*‐test was used to compare the continuous variables. Cochran–Mantel–Haenszel test was performed to determine the age‐specific prevalence of antibiotic use in children with and without SAM. The strength of association was determined by calculating the odds ratios (ORs) and their 95% confidence intervals (CIs). A probability of <0.05 was considered statistically significant. We have performed multivariable logistic regression analysis to investigate the relationship between antibiotic use and the nutritional status of the children.

## Results

Overall, 5279 young infants were included in this analysis. Among them, 257 (4.9%) had SAM. Of the 257 infants, 99 (38.5%) had bilateral pedal oedema. The mean (SD) age of the participants was 4.24 (1.14) months. 41.2% were female, and there was no difference between children with SAM and non‐SAM in terms of sex. The average weight and length were significantly lower in SAM infants compared to non‐SAM infants (4.52 kg vs. 5.88 kg, *P* value < 0.001; 58.8 cm vs. 60.6 cm, *P*‐value < 0.001). Infants with bilateral pedal oedema were not exclusively breastfed compared to infants without oedema, and the difference was statistically significant (97% vs. 78.6%, *P* value < 0.001). In this study, more than half (52%) of the young infants received antibiotics before hospital admission. The use of antibiotics was higher among infants without SAM (52.5%) compared to infants with SAM (42%), and the difference was statistically significant (*P* value = 0.001) (Table 1). No difference was, however, observed in receiving antibiotics among the enrolled infants in terms of sex (male = 52.4% vs. female = 51.4%), EBF status (51.1% vs. 52.2%) and formula feeding status (52.3% vs. 50.9%).

**Table 1:** Baseline characteristics of the participants included in the analysis

Variables	Overall	SAM	non‐SAM	*P* value
	(*n* = 5279)	(*n* = 257)	(*n* = 5022)	SAM vs. non‐SAM
Age in months, mean (SD)	4.24 (1.14)	3.90 (1.22)	4.26 (1.14)	< 0.001
Sex (female), *n* (%)	2176 (41.2)	100 (38.9)	2076 (41.3)	0.44
EBF status (yes), *n* (%)	1109 (21.0)	17 (6.6)	1092 (21.7)	< 0.001
Formula feeding (yes), *n* (%)	3991 (75.6)	234 (91.1)	3757 (74.8)	< 0.001
Weight in kg, mean (SD)	5.81 (1.18)	4.52 (1.03)	5.88 (1.14)	< 0.001
Length in cm, mean (SD)	60.5 (3.88)	58.8 (4.43)	60.6 (3.83)	< 0.001
WLZ, mean (SD)	−0.52 (1.34)	−3.63 (0.642)	−0.43 (1.23)	< 0.001
Antibiotic consumed (yes), *n* (%)	2744 (52)	108 (42)	2636 (52.5)	0.001
Congenital anomaly (yes), *n* (%)	16 (0.3)	7 (2.7)	9 (0.2)	< 0.001
Down's syndrome (yes), *n* (%)	9 (0.2)	5 (1.9)	4 (0.1)	< 0.001
Anuria (yes), *n* (%)	2 (0.0)	1 (0.4)	1 (0.0)	0.10
Septic shock (yes), *n* (%)	13 (0.2)	1 (0.4)	12 (0.2)	0.48
Bilateral pedal oedema (yes), *n* (%)	99 (1.9)	99 (38.5)	0 (0)	—
Jaundice (yes), *n* (%)	1 (0.0)	0 (0)	1 (0.0)	1.00
Maternal use of antibiotics (yes), *n* (%)	13 (0.2)	0 (0)	13 (0.3)	1.00

—, no value; EBF, exclusively breastfed; SAM, severe acute malnutrition; SD, standard deviation; WLZ, weight‐for‐length z‐scores.

### Factors associated with antibiotic use in young infants

Infant's age in months, nutritional status and other variables that were correlated with infant's nutritional status were associated with antibiotic consumption and the associations were statistically significant (Table 2). Multi‐variable logistic regression analysis showed that age in months and the nutritional status of the infants was significantly associated with the use of antibiotics in young infants with diarrhoea. Use of antibiotics increases with age (aOR = 1.11, 95% CI: 1.06, 1.17, *P* value < 0.001), and non‐SAM infants had higher odds of receiving antibiotics (aOR = 1.52, 95% CI: 1.18, 1.97, *P* value = 0.003) before hospital admission compared to infants with SAM. (Table 3).

**Table 2:** Association of antibiotic use with different variables in infants

Variables	Antibiotics	No antibiotics	
	(*n* = 2744)	(*n* = 2535)	*P* value
Age in months, mean (SD)	4.31 (1.11)	4.16 (1.18)	<0.001
Sex (female), *n* (%)	1118 (40.7)	1058 (41.7)	0.46
EBF status (yes), *n* (%)	567 (20.7)	542 (21.4)	0.52
Formula feeding (yes), *n* (%)	2088 (76.1)	1903 (75.1)	0.39
WLZ, mean (SD)	−0.48 (1.30)	−0.57 (1.38)	0.013
Nutritional status (non‐SAM), *n* (%)	2636 (96.1)	2386 (94.1)	0.001
Congenital anomaly (yes), *n* (%)	9 (0.3)	7 (0.3)	0.59
Down's syndrome (yes), *n* (%)	5 (0.2)	4 (0.2)	1.0
Anuria (yes), *n* (%)	1 (0.0)	1 (0.0)	1.0
Septic shock (yes), *n* (%)	8 (0.3)	5 (0.2)	0.585
Bilateral pedal oedema (no), *n* (%)	2706 (98.6)	2474 (97.6)	0.006
Jaundice (yes), *n* (%)	0 (0.0)	1 (0.0)	0.48
Maternal use of antibiotics (yes), *n* (%)	7 (0.3)	6 (0.2)	0.60

EBF, exclusively breastfed; SAM, severe acute malnutrition; SD, standard deviation; WLZ, weight‐for‐length z‐scores.

**Table 3:** Association of antibiotic use with the nutritional status of the infants

Variables	Antibiotic consumed (yes) (*n* = 2744)	Antibiotic consumed (no) (*n* = 2535)	OR (95% CI)	p‐value	aOR (95% CI)	*P* value
Age in months, mean (SD)	4.31 (1.11)	4.16 (1.18)	1.12 (1.07, 1.17)	< 0.001	1.11 (1.06, 1.17)	< 0.001
Sex (female)	40.7%	41.7%	0.96 (0.86, 1.07)	0.46	0.96 (0.86, 1.07)	0.46
Nutritional status (non‐SAM)	96.1%	94.1%	1.52 (1.18, 1.97)	0.001	1.47 (1.14, 1.90)	0.003

aOR, adjusted odds ratio; CI, confidence interval; OR, odds ratio; SAM, severe acute malnutrition; SD, standard deviation.

### Percentages of antibiotic use in young infants

Commonly used antibiotics in infants were azithromycin (13.3%, *n* = 700), ciprofloxacin (7.7%, *n* = 407), erythromycin (7.7%, *n* = 409) and metronidazole (2.6%, *n* = 138) (Table S1). Among all the antibiotics used by the study participants, ceftriaxone (*n* = 53) and cefixime (*n* = 53) had 1% prevalence, whereas flucloxacillin, levofloxacin, meropenem, amoxicillin, ampicillin, amikacin, gentamicin, clarithromycin, doxycycline, ceftazidime, cefuroxime, cefradine, cefaclor, cefpodoxime, ceftibuten, co‐trimoxazole, pivmecillinam, trimethoprim had less than 1% prevalence. Around 19% of the caregivers could not mention the name of the antibiotic received by the infants, and only 1% of young infants received more than one antibiotic. The proportion of ciprofloxacin use prior to hospital admission was more in the non‐SAM group in comparison to malnourished group (7.97% vs. 2.7%), which was statistically significant (*P* value = 0.004).

**Fig 1:**
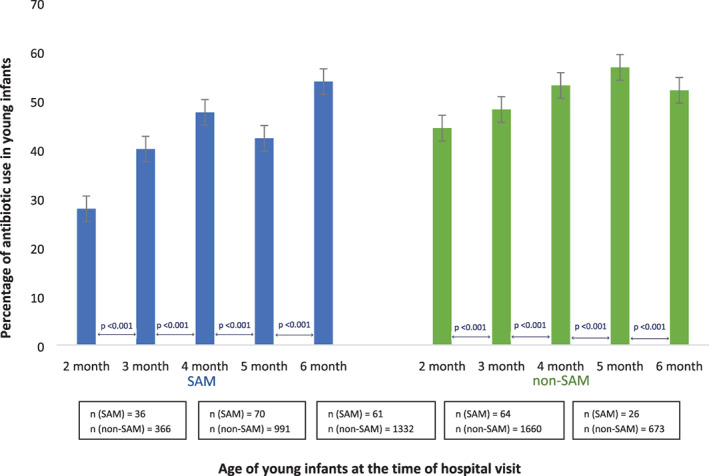
Age‐specific prevalence of antibiotic use in children with and without severe acute malnutrition (SAM).

Figure 1 shows that the prevalence of antibiotic use prior to hospital admission significantly increased with age up to 5 months of age among all the participants and decreased at 6 months of age (*P* value < 0.001). The prevalence of antibiotic use in non‐SAM infants follows a similar trend but was different in SAM children at 5 and 6 months of age. The prevalence of antibiotic use in SAM infants increased with age up to 4 months of age, then decreased at 5 months, but again increased at 6 months of age and the differences were statistically significant (*P* value < 0.001).

## Discussion

The study results show the practice of antibiotic prescribing to infants suffering from diarrhoeal diseases prior to their hospital admission. In a low‐to‐middle income country like Bangladesh, the increased use of antibiotics in such young infants is striking. Among 5279 infants, more than half the participants were prescribed antibiotics. There can be multiple determinants of the higher use of antibiotics in a country like Bangladesh. In low‐income countries, medicines are available to the general population from diversified sources including hospitals, local pharmacies, licensed drug stores, roadside drug shops and unskilled practitioners or traditional healers.^18^, ^19^ Getting access to antimicrobials without a prescription is also not difficult, although such practice is considered illegal.^19^ A review article found lack of appropriate knowledge among practitioners, mistrust in or delayed lab results, health‐care practitioner's desire to be on the safe side, to meet patient demand and traditional and social beliefs on antibiotic use to be some of the key determinants, which may have influenced antibiotic prescribing practice.^20^ It is not surprising that different authors tried to advocate that different levels of training and apprehension of antibiotic prescribing contribute to different types of prescribing.^19^, ^21^ However, this might not be the case always. For example, metronidazole prescribing rates were equal among the doctors and medical assistants in a Bangladeshi study.^22^ In a Peruvian's study, it has been seen that although physicians had adequate knowledge regarding diarrhoeal management, they prescribed antimicrobials to almost all the patients. Patient's confirmation about the absence of blood did not stop them from prescribing antimicrobials without confirmatory investigations.^23^ In an Indonesian study, both the public and private practitioners stated that viral illness is more common than bacterial origin but when it came to advising drugs, more than half of the practitioners prescribed antibiotics in each group for diarrhoeal treatment.^24^ Patient's demand to be treated with a stronger antibiotic also influences the prescriber's decision.^24^ Empirical antibiotic prescription is also popular in low‐socio‐economic countries where a diagnostic approach to disease is considered unnecessary and costly by patients. Only in the event of deterioration of disease after the initial antibiotic treatment, laboratory investigations are performed.^25^ People sometimes prefer purchasing drugs directly from drug stores without any prior consultation with a registered physician. This happens mainly because people think it is profitable to buy medicine first hand rather than getting a medical consultation.^26^ Insufficient knowledge of drug sellers, acting on customer demand and lack of law enforcement are found to be some of the key determinants that increase indiscriminate antibiotic dispensing.^20^ All these facts give a reasoning behind the increased use of antibiotics among infants due to diarrhoeal illnesses before visiting Dhaka Hospital.

We observed antibiotic intake was significantly more in non‐SAM infants in contrast to the malnourished group. Malnutrition itself is a critical condition that makes it difficult for the children to recover and is a bad prognostic factor.^27^ Among all other co‐morbidities, diarrhoea is found to be the most predominant to affect SAM children.^28^ Dehydrating diarrhoea is difficult to manage in children with SAM as relative immunodeficiency and delayed intestinal repair play a role in respective fluid and electrolyte loss mechanisms.^27^ As SAM children tend to get in the more complicated clinical condition, it may be so that antibiotic prescribers did not recommend outpatient treatment, rather they advised them to get facility‐based management, whereas the non‐SAM children were the more targeted group for at‐home treatment with antibiotics. One possible explanation of non‐SAM children having being prescribed more antibiotics could be this.

Different forms of malnutrition are associated with socio‐economic inequality.^29^ Wasted children are more likely to come from lower socio‐economic status.^29^ Another hypothesis for the higher usage of antibiotics among the non‐SAM group could be that infants with SAM could not afford the antibiotics, whereas non‐SAM infants could. As a result, SAM infants visited the hospital without taking any further outside medication as opposed to the non‐SAM group who consumed the antimicrobials prior to coming to the hospital.

Antimicrobials can be used dependably for cases with bloody diarrhoea suspecting probable shigellosis and cholera‐like illness. If amoebiasis or giardiasis has been confirmed by investigation, it is indicated to be treated by metronidazole.^6^ In the past, drugs like ampicillin, chloramphenicol and co‐trimoxazole were used to treat diarrhoeal diseases, but our study findings show more consumption of different antibiotics. In Bangladesh, azithromycin, ciprofloxacin and metronidazole are some of the most commonly used antimicrobials in the populace.^30^ Also, the WHO suggests macrolide and fluoroquinolone for diarrhoeal management in children when it is indicated.^6^ Diarrhoea treatment protocol for adults also suggests practicing azithromycin, ciprofloxacin and metronidazole in cases where they are necessary.^31^ We can speculate that this information is also influencing the changing pattern and indiscriminate use of antibiotics in young infants. All of these factors may be contributing to the fact that macrolides and fluoroquinolones had such high prevalence in our study. Our findings included that azithromycin, ciprofloxacin, cefixime and metronidazole consumption was more among non‐SAM infants among which only ciprofloxacin was found to be significantly associated with non‐SAM infants compared to SAM infants.

In our study population, antibiotic consumption has been increasing with age, which needs further evaluation. We can theorise that as rotaviral diarrhoeal episodes tend to increase with age in children under 1 year of age, this factor could be contributing to this finding.^32^ Another hypothesis could be that, in diarrhoea endemic countries like Bangladesh, it is very common for children to get infected several times as poor hygiene practice, lack of sanitation, mother's insufficient knowledge in prevention of diarrhoea and inept dietary practices are potential risk factors and the enteropathogen burden is visible from the first month of life.^33^, ^34^ In response to this, caregivers might have allowed their infants to ingest antibiotics even if they were hesitant during previous episodes when the child was younger. A study conducted in Australia showed the likelihood that an antibiotic is going to be prescribed increases with the advancement of age, which is also evident with the findings from our study.^35^ Regardless, further research is needed to explore the relationship between antibiotic consumption and the age of young infants in Bangladesh.

The percentage of exclusively breastfed (EBF) infants was 21% in our study, which is relatively lower than the country prevalence of 65%.^11^ There may be several factors for this variation. According to WHO, EBF children will not receive any solids or liquids apart from breastmilk except medicines.^36^ The study has used the WHO definition to verify EBF. The country survey data of EBF were collected by 24‐h recall method, which did not take into account prelacteal feeding of the babies, and Bangladesh has a high prevalence of prelacteal feeding.^11^, ^37^ Also, a study conducted in 2014 found a lower percentage (36%) of EBF compared to the country percentage (55%) of 2014.^38^ The study also excluded infants younger than 2 months and EBF rates are seen to be higher in the early days of life. Furthermore, the population of our study was different as they were diarrhoeal children.

For the pressing need to refine antibiotic use and fight AMR, WHO has adopted a global action plan on AMR (GAP) in collaboration with Food and Agriculture Organisation of the United Nations and World Organisation for Animal Health (OIE) at the World Health Assembly in 2015.^39^ Their primary goal was to treat and prevent infectious diseases with safe medications used responsibly. The inappropriate use of antimicrobials in low‐to‐middle income countries has been recognised by WHO resulting in the promotion of a range of policies. In 2015, WHO launched the GLASS programme (Global Antimicrobial Resistance Surveillance System) across countries to support regional, national and global efforts to tackle AMR and irresponsible consumption of antibiotics.^40^ Bangladesh has initiated Antimicrobial Consumption Monitoring Taskforce with support of WHO but is still developing systems to monitor antibiotic use.^39^

Lessons can be learnt from different environments across the globe about the potential adverse effects of AMR. Different studies from Mexico, Brazil, China, Thailand and France have reported increased mortality rate of intensive care unit patients which is associated with AMR.^41^ Other than AMR, dysbiosis of the gut microbiota and systemic fungal infection are important factors to discuss, which are associated with inappropriate antibiotic use. Intestinal microbiota intricates metabolic functions, regulates energy intake, affects nutritional development, modulates the immune system, affects intestinal mucosal structure and helps in developing neurological functions of the body.^42^ Disruption of this complex ecosystem of the body can be associated with intestinal and extraintestinal diseases as well as malnutrition.^14^, ^42^ Inappropriate or excessive antibiotic therapy that disrupts the normal flora on the mucosal surfaces and skin can be associated with systemic fungal infection.^43^ Extreme age and broad‐spectrum antimicrobial use are risk factors for systemic candidiasis.^44^ Paediatric intensive care unit patients are common victims of this condition.^44^ To promote rational use of antibiotics, necessary interventions need to be advocated. A multidisciplinary national body to implement policies on rational medicine use needs to be established. Other key interventions like utilising proper clinical guidelines, formulating national essential medicines list, raising public awareness against AMR through media, practicing appropriate law and regulation, compulsory training of health‐care professionals and medicine traders are suggested.

### Limitations of the study

Our study has some limitations. Data were collected from the screening records, which held limited information restricting us to further analyse the possible relationship of antibiotic consumption with different variables, for example; socio‐economic status, birth history of the infants describing prematurity and low birthweight, previous hospital admission, the prescribed antibiotic was from a registered physician or local drug dispenser. The data derived were in a cross‐sectional manner, which limited us in investigating the trend of antibiotic use with age among young infants, which would have been possible if we possessed longitudinal data. Inadequate morbidity information confined us from investigating the rationality of antibiotic use in these younger infants, such as the history or clinical evidence related to watery or bloody diarrhoea, dehydration status. We did not follow‐up the participants as this was a cross‐sectional study and we consider it as a limitation. However, this does not affect the primary objective of the study, which is to investigate the prevalence of antibiotic use among diarrhoeal infants in this particular age group before seeking facility‐based management.

## Conclusions

The study illustrates the higher trend of antibiotic use among young infants, which is rather alarming. This further begets the question of what should be done to control this rampant use of antibiotics in diarrhoeal management. Excessive antibiotic exposure may bring about further complications, for example, AMR; a worrisome threat that is slowly spreading over the world. Policymakers of the country also need to step up and formulate new policy and guideline to control this irrational antibiotic prescribing practice in Bangladesh and elsewhere.

## Supporting information


**Table S1.** Six most frequently consumed antibiotics; overall and in infants with and without SAM

## Data Availability

Data related to this manuscript are available upon request and for researchers who meet the criteria for access to confidential data may contact with Armana Ahmed (armana@icddrb.org) to the Research Administration of International Centre for Diarrhoeal Disease Research, Bangladesh (http://www.icddrb.org/).
