# Supplementary material for: Antibiotic exposure among young infants suffering from diarrhoea in Bangladesh
Source: J Paediatr Child Health. 2020 Oct 27;57(3):395–402. doi: 10.1111/jpc.15233 (PMC8048795; doi:10.1111/jpc.15233)
Supplement: Supplementary file 1 — Table S1. Six most frequently consumed antibiotics; overall and in infants with and without SAM [file JPC-57-395-s001.docx]

Supporting information: Six most frequently consumed antibiotics; overall and in infants with and without SAM

| Name of the antibiotic | Overall  (n = 5279) | SAM  (n = 257) | non-SAM  (n = 5022) |
| --- | --- | --- | --- |
|  |  |  |  |
| Azithromycin, n (%) | 700 (13.3%) | 27 (10.5%) | 673 (13.4%) |
| Ciprofloxacin, n (%) | 407 (7.7%) | 7 (2.7%) | 400 (7.97%) |
| Erythromycin, n (%) | 409 (7.7%) | 21 (8.2%) | 388 (7.7%) |
| Ceftriaxone, n (%) | 53 ( 1.0%) | 5 (1.95%) | 48 (0.96%) |
| Cefixime, n (%) | 53 (1.0%) | 1 (0.4%) | 52 (1.04%) |
| Metronidazole, n (%) | 138 (2.6%) | 5 (1.95%) | 133 (2.7%) |
